# Exploring immune-related signatures for predicting immunotherapeutic responsiveness, prognosis, and diagnosis of patients with colon cancer

**DOI:** 10.18632/aging.204134

**Published:** 2022-06-20

**Authors:** Lichao Cao, Ying Ba, Jin Yang, Hezi Zhang

**Affiliations:** 1 Provincial Key Laboratory of Biotechnology of Shaanxi Province, Northwest University, Xi’an, China; 2 Key Laboratory of Resource Biology and Biotechnology in Western China, Ministry of Education, School of Life Sciences, Northwest University, Xi’an, China; 3 Shenzhen Nucleus Gene Technology Co., Ltd., Shenzhen, China

**Keywords:** colon cancer, tumor immune microenvironment, prognosis, diagnosis, immunotherapeutic responsiveness

## Abstract

The present study focused on identifying the immune-related signatures and exploring their performance in predicting the prognosis, immunotherapeutic responsiveness, and diagnosis of patients with colon cancer. Firstly, the immunotherapeutic response-related differential expressed genes (DEGs) were identified by comparing responders and non-responders from an anti-PD-L1 cohort using the edgeR R package. Then, the immunotherapeutic response related DEGs was intersected with immune-related genes (IRGs) to obtain the immunotherapeutic response and immune-related genes (IRIGs). Then, an immunotherapeutic response and immune-related risk score (IRIRScore) model consisting of 6 IRIGs was constructed using the univariable Cox regression analysis and multivariate Cox regression analysis based on the COAD cohort from the cancer genome atlas (TCGA) database, which was further validated in two independent gene expression omnibus database (GEO) datasets (GSE39582 and GSE17536) and anti-PD-L1 cohort. A nomogram with good accuracy was established based on the immune-related signatures and clinical factors (C-index = 0.75). In the training dataset and GSE39582, higher IRIRScore was significantly associated with higher TMN and advanced pathological stages. Based on the anti-PD-L1 cohort, patients who were sensitive to immunotherapy had significantly lower risk score than non-responders. Furthermore, we explored the immunotherapy-related signatures based on the training dataset. Kaplan-Meier curve revealed a high level of T cells regulatory (Tregs) was significantly related to poor overall survival (OS), while a high level of T cells CD4 memory resting was significantly related to better OS. Besides, the TMB value of patients in the high-risk group was significantly higher than those in a low-risk group. Moreover, patients in the high-risk group had significantly higher expression levels of immune checkpoint inhibitors. In addition, the immune-related signatures were applied to establish prediction models using the random forest algorithm. Among them, *TDGF1* and *NRG1* revealed excellent diagnostic predictive performance (AUC >0.8). In conclusion, the current findings provide new insights into immune-related immunotherapeutic responsiveness, prognosis, and diagnosis of colon cancer.

## INTRODUCTION

Colon cancer is one of the most malignant tumors with high mortality worldwide despite advancements in tumor screening, early diagnosis, and treatment [1]. In recent years, increasing studies explored the functions of tumor immune microenvironment, which may play important roles in tumorigenesis [2]. Moreover, numerous studies have identified immune-related signatures in the tumor microenvironment for predicting the prognosis for various cancers, including colon cancer [3–5].

Immunotherapy, especially immune checkpoint inhibitors (ICIs), focuses on harnessing the host immune system to combat tumor progression and metastasis and achieve the goal of controlling and eliminating tumors, which has revolutionized the treatment for colon cancer [6]. However, the responsiveness rate to ICIs rarely exceeds 20%, which depends on several key factors, including mutational load, tumor-infiltrating lymphocytes, and regulatory checkpoint receptors [7]. However, the research on molecular markers for predicting the immunotherapeutic responsiveness of colon cancer patients is limited.

Therefore, it is critical to explore effective and robust novel molecular signatures for predicting the immunotherapeutic responsiveness, prognosis, and diagnosis of patients with colon cancer. The present study focused on the immune-related signatures identified in the anti-PD-L1 cohort and systematically explored their performance in predicting the prognosis, immunotherapeutic responsiveness, and diagnosis of patients with colon cancer.

## MATERIALS AND METHODS

### Acquiring and preprocessing the relevant data

An anti-PD-L1 cohort (IMvigor210) was obtained using the IMvigor210CoreBiologies R package [8], which was treated with atezolizumab (anti-PD-L1 agent); only the samples with immunotherapeutic response information were included. The samples that displayed the complete response (CR) or partial response (PR) were categorized as responders and the samples that displayed stable (SD) or progressive disease (PD) were categorized as non-responders. In addition, the scores (estimate, stromal, and immune) of each sample were calculated using the estimate R package [9]. The differences in the clinical features and the scores between responders and non-responders were statistically compared using the Wilcoxon test, and the detailed information is shown in Table 1 and Supplementary Figure 1A–1C.

**Table 1:** The detailed information on Anti-PD-L1 cohort.

	**CR/PR**	**SD/PD**	***P*-value**
	**(*N* = 68)**	**(*N* = 230)**	
**Sex**			
F	11 (16.2%)	54 (23.5%)	0.125
M	57 (83.8%)	176 (76.5%)	
**Baseline.ECOG.Score**			
0	37 (54.4%)	84 (36.5%)	0.0313
1	27 (39.7%)	138 (60.0%)	
2	4 (5.9%)	8 (3.5%)	
**sizeFactor**			
Mean (SD)	1.12 (0.419)	1.06 (0.360)	0.393
Median (Min, Max)	1.11 (0.306, 1.95)	1.02 (0.264, 1.97)	
**OS**			
Alive	63 (92.6%)	46 (20.0%)	0.125
Death	5 (7.4%)	184 (80.0%)	
**OS.time**			
Mean (SD)	590 (94.1)	271 (194)	<0.001
Median (Min, Max)	618 (278, 734)	214 (5.91, 715)	
**FMOne.mutation.burden.per.MB**			
Mean (SD)	16.9 (13.3)	8.61 (6.26)	<0.001
Median (Min, Max)	14.0 (1.00, 62.0)	7.00 (0, 44.0)	
Missing	7 (10.3%)	57 (24.8%)	
**Neoantigen.burden.per.MB**			
Mean (SD)	2.46 (2.34)	1.06 (1.09)	<0.001
Median (Min, Max)	1.80 (0.216, 11.7)	0.725 (0.0392, 6.20)	
Missing	15 (22.1%)	67 (29.1%)	

Abbreviation: OS: overall survival.

A total of 471 COAD samples were downloaded from UCSC Xena platform (https://xenabrowser.net/datapages/), including the mRNA expression data, mutation profiling data, and clinical and survival information. Subsequently, the samples (*n* = 471) were divided into the normal group (*n* = 39) and tumor group (*n* = 432); the tumor group was used as the training dataset, and the detailed information is listed in Supplementary Table 1.

Two independent GEO datasets (GSE39582 and GSE17536), considered testing datasets, were downloaded from the GEO database (https://www.ncbi.nlm.nih.gov/geo/). The GSE39582 included 556 colon cancer patients, and GSE17536 included 177 colon cancer patients, with available clinical and survival information. The detailed information was shown in Supplementary Tables 2 and 3.

A total of 1509 IRGs were obtained from the ImmPort database (https://immport.niaid.nih.gov/).

### Identification of immune-related genes from the anti-PD-L1 cohort

The edgeR R package [10] was adopted to compare the gene expression differences between the responders and non-responders. Genes that met the cut-off criteria of false discovery rate (FDR) <0.05 and |log2 fold change (FC)|>1 were considered immunotherapeutic response related DEGs, and then intersected with IRGs to obtain the IRIGs. Subsequently, the Gene ontology (GO) functional, Kyoto Encyclopedia of Genes and Genomes (KEGG) enrichment analysis was performed on IRIGs using the clusterProfiler R package [11].

### Construction of estimate-stromal-immune scores model

The ESTIMATE algorithm calculated the immune, stromal and estimate scores for each colon cancer sample of the TCGA-COAD cohort [9]. Then, the colon cancer patients were divided into the high-score and low-score groups based on the median levels of the estimate, stromal, and immune scores, respectively. The association between the estimate-stromal-immune scores and OS was analyzed using the Kaplan-Meier curve. The pROC R package was used to plot the receiver operating characteristic (ROC) curves to evaluate the predictive capacities of the estimate-stromal-immune scores model.

### Identification and validation of immune-related signatures for predicting immunotherapeutic responsiveness and prognosis of patients with colon cancer

The univariable Cox regression analysis was performed on the IRIGs using the training dataset with *P* < 0.05 as the criteria to identify the prognostic signature. Subsequently, we established the IRIRScore model for predicting the prognosis of colon cancer patients using the multivariate Cox regression analysis and the coefficient of the prognostic indicator was obtained. The IRIRScore for each patient was calculated according to the formula:


$$\text{IRIRScore}=\sum Cox\;coefficient\;of\;gene\;\chi^{i}\times scale\;expression\;value\;of\;gene\;\chi^{i}$$

Moreover, two independent GEO datasets with accession numbers GSE39582 and GSE17536 and anti-PD-L1 cohort were used to further validate the prognostic signatures of the constructed IRIRScore model. Besides, the Kruskal-Wallis test was used to compare the difference in IRIRScore between responders and non-responders in the anti-PD-L1 cohort.

In addition, a nomogram was designed to visualize the prognostic value of different patients’ features, including IRIRScore, StromalScore, ImmuneScore, age, gender, and tumor stage. The calibration curves were plotted to evaluate the predicted probabilities compared to the ideal predictive line using the rms R package. Moreover, the hazard ratios of the patients’ features were illustrated by a forest plot.

### Statistical analysis

To verify and access the predictive capabilities of the constructed IRIRScore model, the ROC curves and Kaplan-Meier curves were plotted using the training dataset (tumor samples in TCGA-COAD cohort) and testing dataset (GSE39582, GSE17536, and anti-PD-L1 cohort). The optimal cutoff value for grouping patients were chosen at the points of the ROC curves where the difference between true positive and false positive was the most significant. Additionally, the associations between the risk score level and clinical features, including tumor stages and microsatellite status, were performed using the Kruskal-Wallis test.

### Exploring potential immunotherapy related signatures in the tumor microenvironment of colon cancer

Firstly, the training dataset was divided into a high-risk group and a low-risk group based on the constructed IRIRScore model. Moreover, the proportions of 22 types of tumor-infiltrating immune cells of each sample in the TCGA-COAD cohort was estimated using the CIBERSORT algorithm [12]. Then, the statistical difference in immune landscape between the high-risk and low-risk score groups was compared using an unpaired *t*-test. The association between the significant differential immune cell types (*P-value* < 0.01) and OS were further accessed using Kaplan-Meier curve. Moreover, the correlations between tumor-infiltrating immune cells and IRIRScore were analyzed using the cor.test in R software (version 4.0.2, http://www.R-project.org).

In addition, the maftools R package was utilized to calculate the TMB value and visualize the mutation profiles of the high- and low-risk groups [13]. Subsequently, the TMB value between high- and low-risk groups was compared by the Kruskal-Wallis test, and the differences in the mRNA levels of immune checkpoints and their ligands between the high- and low-risk groups were statistically compared using the Wilcoxon test.

### Preliminary exploration of IRIGs as predictive diagnostic indicators base on TCGA-COAD cohort

To explore the potential application of the IRIGs in the diagnosis prediction of colon cancer, we compared the expressions of the IRIGs between tumor and normal samples using the Wilcoxon test. Moreover, the IRIGs were employed as independent biomarkers to establish diagnosis prediction models using Random Forest (RF) algorithm. Besides, we tried to use the IRIGs as keywords to search immunohistochemical images in the Human Protein Atlas (HPA) (https://www.proteinatlas.org/), which may experimentally verify our findings.

### Data availability statement

The immunotherapeutic cohort (IMvigor210) was available according to the guideline on http://research-pub.gene.com/IMvigor210CoreBiologies using the IMvigor210CoreBiologies R package. The TCGA-COAD cohort was available in the UCSC Xena (https://xenabrowser.net/datapages/). GSE39582 and GSE17536 were downloaded from NCBI-GEO database (https://www.ncbi.nlm.nih.gov/geo/).

## RESULTS

### Identification of IRIGs

**Figure 1:**
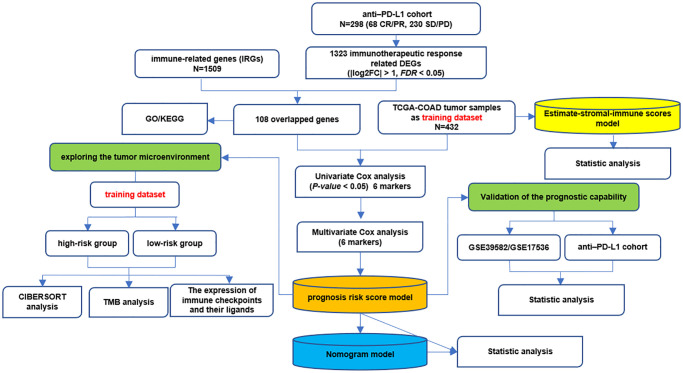
The whole analysis flow of this study.

**Figure 2:**
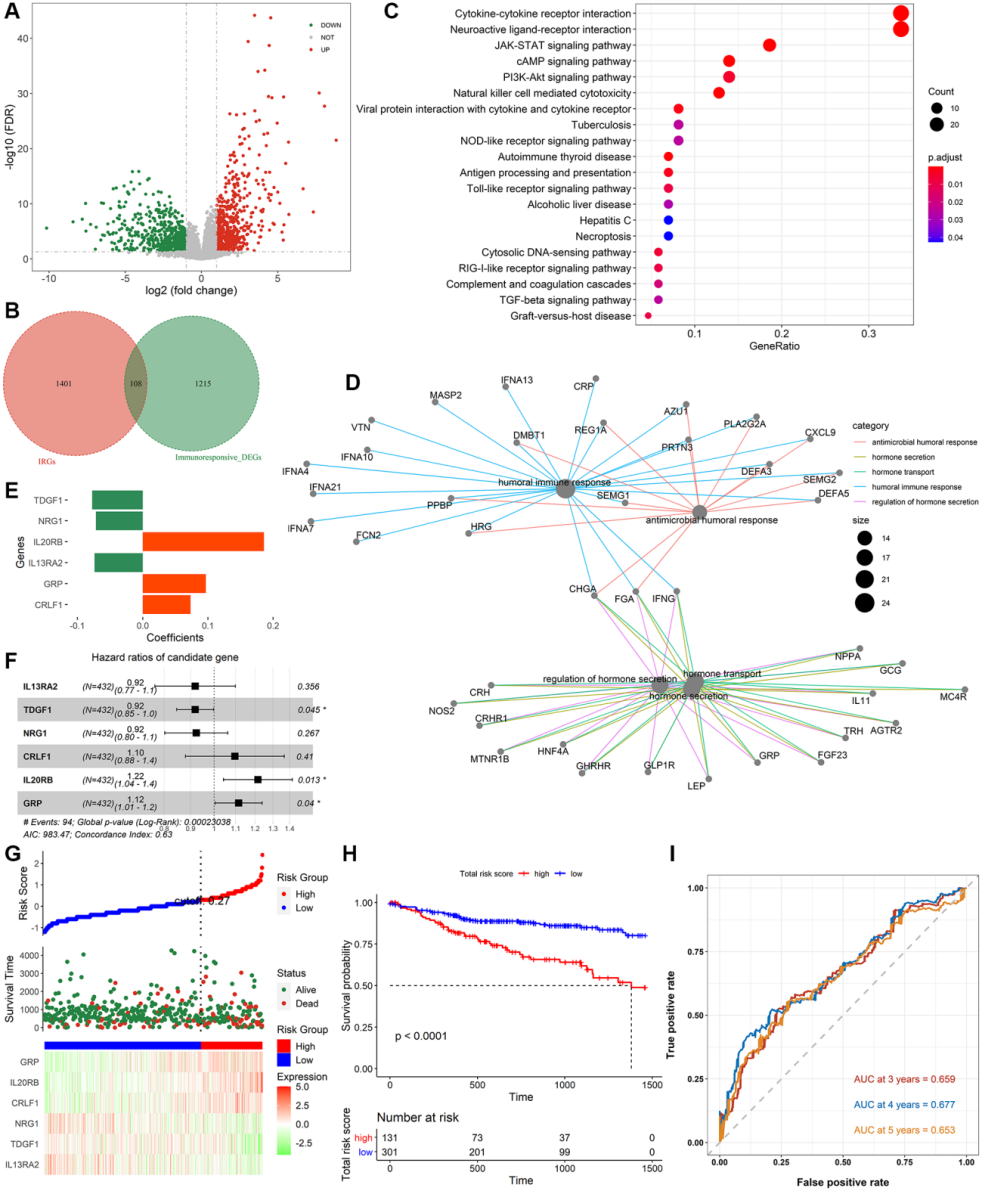
**Identification of immune-related signatures and construction of IRIRScore prognostic model.** (**A**) Volcano plot on immunotherapeutic response related DEGs between the responders and non-responders in anti-PD-L1 cohort. The green dots represent downregulated genes, while the red dots represent upregulated genes. (**B**) Venn diagram of the intersection between the immunotherapeutic response related DEGs and IRGs. (**C**) KEGG pathway enrichment analysis of IRIGs. (**D**) GO analysis of IRIGs. (**E**) The coefficients of the 6 IRIGs related to prognosis of colon cancer. (**F**) Forest plot showing the HR of each IRIG. (**G**) The distribution of samples in the high- and low risk score groups and their relationship with OS, and the expression pattern of 6 prognostic signatures in high- and low risk score groups. (**H**) Kaplan-Meier curve exhibited that OS of patients in the low-risk score group was significantly higher than those in the high-risk score group. (**I**) Time-dependent ROC curve analysis of the IRIRScore model.

Figure 1 shows the whole analysis flow of this study. 1323 immunotherapeutic response-related DEGs (634 upregulated and 689 downregulated) were identified by comparing responders and non-responders in the anti-PD-L1 cohort (Figure 2A, Supplementary Table 4). After the intersection with 1509 IRGs, 108 IRIGs was obtained (Figure 2B, Supplementary Table 5). KEGG analysis results revealed that IRIGs were remarkably enriched in terms associated with Cytokine-cytokine receptor interaction, Neuroactive ligand-receptor interaction, JAK-STAT signaling pathway, cAMP signaling pathway, PI3K-Akt signaling pathway, and so on (Figure 2C). Furthermore, the GO enrichment analysis indicated that IRIGs mapped to hormone-related terms, including humoral immune response, antimicrobial humoral response, regulation of hormone secretion, and hormone transport (Figure 2D).

### Construction of immune-related prognostic models

The AUC of estimate-stromal-immune scores model was less than 0.6 (ESTIMATEScore: 0.5; StromalScore: 0.513; ImmuneScore: 0.519, Supplementary Figure 1D), indicating its poor performance for predicting the prognosis of colon cancer. Additionally, the Kaplan-Meier curves revealed that the association between the scores (estimate, stromal, or immune) and OS were not significant (ESTIMATEScore: *P* = 0.61, StromalScore: *P* = 0.47, ImmuneScore: *P* = 0.94, Supplementary Figure 1E–1G).

**Figure 3:**
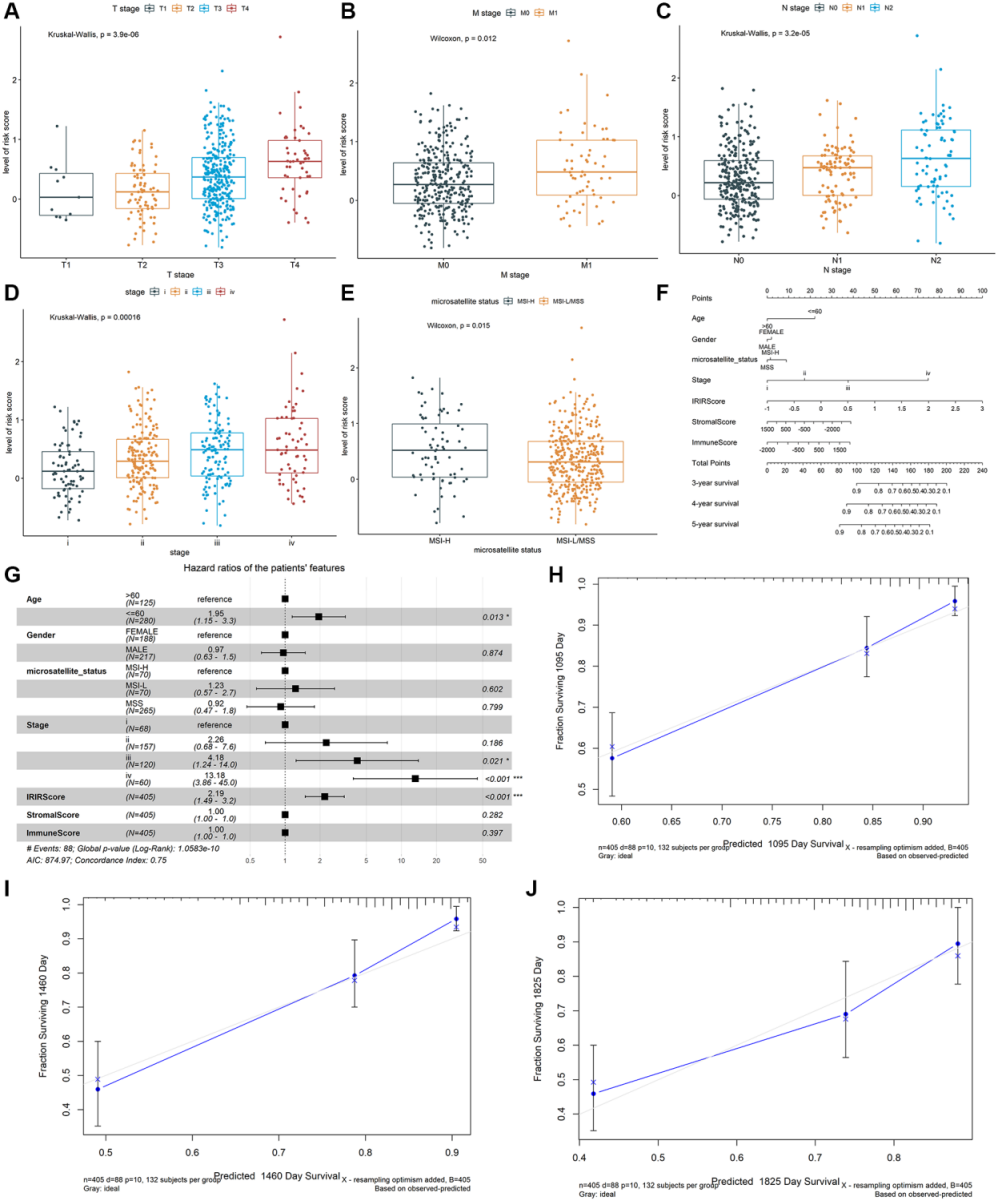
The association between the IRIRScore and the clinical-pathological characteristics, including (**A**) T stages, (**B**) M stages, (**C**) N stages, (**D**) advanced pathological stages, and (**E**) microsatellite status Construction and validation of a nomogram. (**F**) Nomogram to predict the probability of OS in 3, 4 and 5 years for colon cancer. (**G**) Forest plots showing the associations between patient's characteristics and OS. (**H**–**J**) Calibration plot of the nomogram to predict the probability of OS at 3, 4 and 5 years.

Therefore, effective prognostic signatures for patients with colon cancer needed to be explored. Based on the training dataset, the univariate and multivariate Cox regression analyses were performed on the IRIGs, and 6 genes were found to be significantly related to OS status (Table 2). The coefficient and hazard ratio (HR) of each prognostic indicator is shown in Figure 2E and 2F, respectively. *TDGF1*, *IL13RA2*, and *NRG1* are protective factors (HR <1), while *CRLF1*, *GRP*, and *IL20RB* are risk factors (HR >1). Then, the IRIRScore of each patient was calculated according to the formula described in the Materials and methods section. The scatter diagram in Figure 2G displayed that the survival time of patients in the low-risk group was longer than that in a high-risk group, which was also significantly supported by Figure 2H (*P* < 0.0001). The heatmap in Figure 2G reveals that the expression of risk factors was low in the low-risk group and high in the high-risk group, while the trend of protective factors was the opposite. The AUC of IRIRScore model at 3, 4, and 5 years of OS are 0.659, 0.677, and 0.653, respectively (Figure 2I). Patients with larger tumor sizes yielded higher IRIRScore than those with small tumor size (Kruskal-Wallis test, *P* = 3.9e^−6^, Figure 3A). Patients with metastases yielded higher IRIRScore than those without metastases (Kruskal-Wallis test, *P* = 0.012, Figure 3B). Patients with more distant or more lymph nodes are involved in patients with higher IRIRScore (Kruskal-Wallis test, *P* = 3.2e^−5^, Figure 3C). Patients with advanced stage yielded higher IRIRScore than those with early stage (Kruskal-Wallis test, *P* = 0.00016, Figure 3D). Figure 3E reveals that the patients with higher IRIRScore yielded more instability of the microsatellite status (Kruskal-Wallis test, *P* = 0.015, Figure 3E).

**Table 2:** The detailed information on the identified prognostic IRIGs.

**Gene Name**	**HR**	**HR.95L**	**HR.95H**	***p*-value**
IL13RA2	0.859707917	0.753442337	0.980961205	0.024735341
TDGF1	0.916176837	0.845736901	0.992483591	0.031968386
NRG1	0.878737983	0.786729291	0.981507172	0.021978491
CRLF1	1.242539007	1.029878446	1.499112045	0.023368342
IL20RB	1.261588541	1.097635605	1.450030992	0.001069679
GRP	1.126701281	1.019440489	1.245247555	0.019429694

Abbreviation: HR: hazard ratio.

### Validation and assessment of the identified immune-related signatures in predicting prognosis and immunotherapeutic responsiveness

To verify whether the identified immune-related signatures were suitable for different cohorts, The IRIRScore of each patient in two independent GEO datasets (GSE39582 and GSE17536), and the anti-PD-L1 cohort was calculated based on the same method used in the training dataset. Based on the testing datasets, the OS rate of patients in the high-risk group was significantly lower than that in low-risk group (*P* < 0.0001, Supplementary Figure 2A, Supplementary Figure 3A, and 3C), and the areas under the curves (AUCs) of IRIRScore model were calculated (GSE39582: 0.623 at 3-years, 0.607 at 4-years, and 0.563 at 5-years, Supplementary Figure 2B; GSE17536: 0.656 at 3-years, 0.733 at 4-years, and 0.657 at 5-years, Supplementary Figure 3B; Anti-PD-L1 cohort: 0.645, Supplementary Figure 3D).

Based on the testing data from GSE39582, the Kruskal-Wallis test revealed that higher IRIRScore was associated with higher T stage (*P* = 0.00038), metastasis (*P* = 0.011), N stages (*P* = 0.0015), and advanced pathological stage (*P* = 0.021) (Supplementary Figure 2C–2F).

In addition, based on the anti-PD-L1 cohort, we found that patients sensitive to immunotherapy had significantly lower risk scores than non-responders (*P* = 7.1e^−5^, Supplementary Figure 3E), indicating that responders will have better OS than non-responders.

### Construction and validation of the nomogram

A prognostic nomogram was constructed to improve the accuracy of the performance of the IRIRScore model and to provide a quantitative and visual method for predicting 3-, 4-, and 5-years OS probability of patients with colon cancer. In the nomogram, it is easy to estimate the score for each variable on the point scale and calculate the probability of survival at 3-, 4-, 5-years (Figure 3F). Compared with the other variables, IRIRScore had the maximum score points. The forest plot shows that the variables, including the age (>60), advanced pathological stage (III and IV) and IRIRScore are significantly associated with OS (*P* < 0.05, Figure 3G), but the microsatellite status, StromalScore, and ImmuneScore are not (*P* > 0.05).

To verify the predictive performance of the nomogram, we plotted the calibration curves and observed that the predictive curves were close to the ideal curve (Figure 3H–3J). Moreover, the predictive accuracy (C-index) of the nomogram has been dramatically improved from 0.63 to 0.75, indicating good functioning.

### Exploring potential immunotherapy related signatures

**Figure 4:**
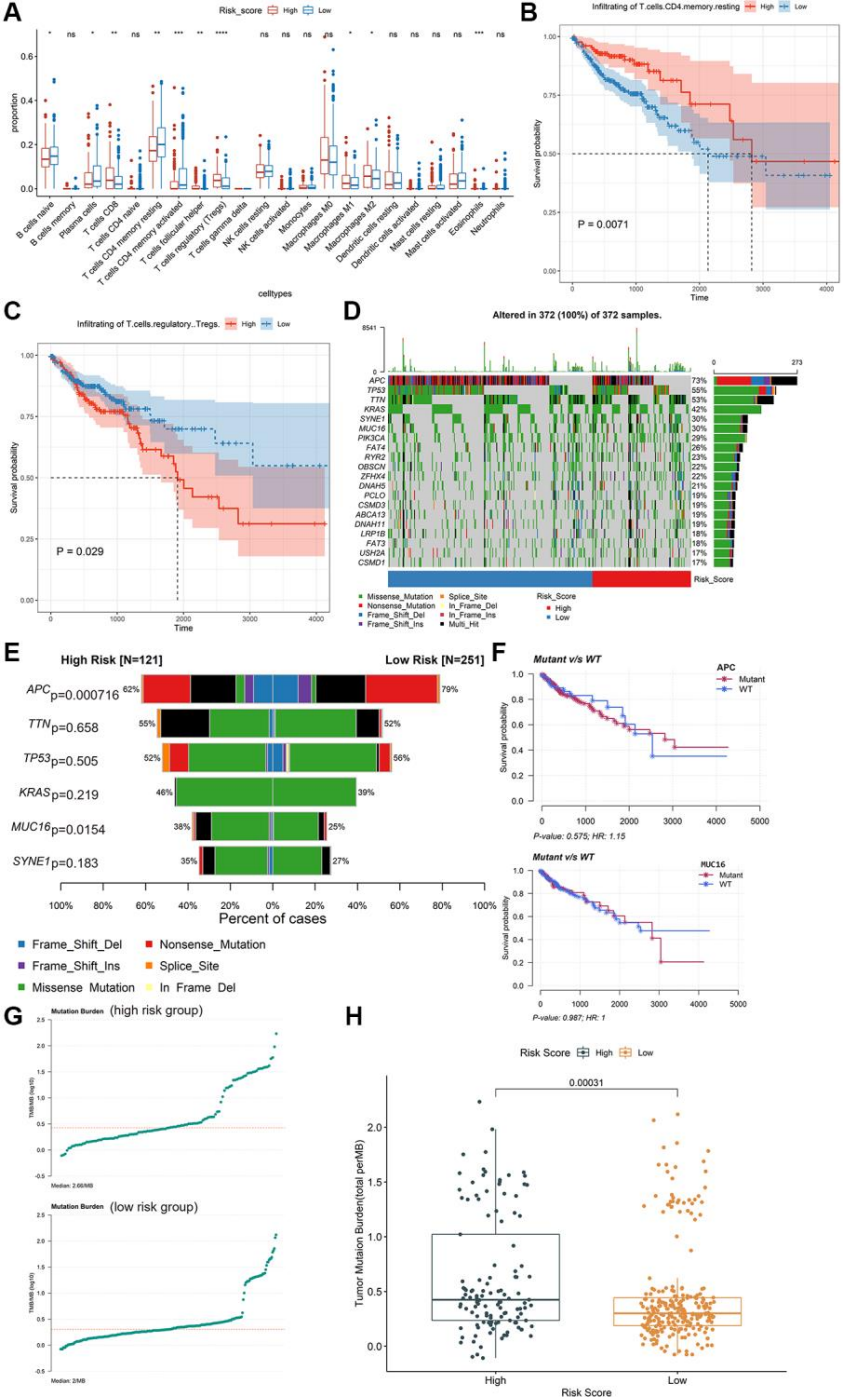
**Exploring potential immunotherapy-related signatures.** (**A**) Comparison of the differences in the proportions of immune cells between low-risk group and high-risk group using the Kruskal-Wallis test. The values of *P* were labelled above each boxplot with asterisks. (^*^P < 0.05, ^**^P < 0.01, ^***^P < 0.001, ^****^P < 0.0001). (**B** and **C**) The Kaplan-Meier analysis of the associations between the level of T cells regulatory (Tregs) and T cells CD4 memory resting with patients’ OS. (**D**) The mutation profiles of the high-risk and low-risk groups. (**E**) Comparison of the mutation rate between high-risk group and low-risk group. (**F**) The association between the mutation status of *APC* and *MUC16* and patients’ OS. (**G**) The TMB profiles of the low-risk group and high-risk group. (**H**) Comparison of the difference in TMB between high-risk and low-risk groups.

We estimated the proportions of 22 types of immune cells in patients with colon cancer in the training dataset using the CIBERSORT method. The profiles of high-risk group and low-risk group were shown in Supplementary Figure 3F and 3G. Subsequently, the composition of immune cell types between the high-risk and low-risk groups was compared using the Kruskal-Wallis test. Moreover, remarkable differences were found in T cells CD8, T cells CD4 memory resting, T cells CD4 memory activated, T cells follicular helper, T cells regulatory (Tregs), and Eosinophils (*P* < 0.01, Figure 4A). The Kaplan-Meier curve revealed that high level of T cells regulatory (Tregs) was significantly related to poor OS (*P* = 0.029, Figure 4C), while a high level of T cells CD4 memory resting was significantly related to better OS (*P* = 0.0071, Figure 4B). 10 of the 22 types of tumor-infiltrating immune cells were significantly related to IRIRScore (*P* < 0.05, Supplementary Figure 4). Among them, T cells regulatory (Tregs), Eosinophils, Macrophages M1, T cells follicular helper, T cells CD8, and Macrophages M2 were positively related to IRIRScore, while Dendritic cells activated, Plasma cells, T cells CD4 memory activated, and T cells CD4 memory resting were negatively related to IRIRScore.

**Figure 5:**
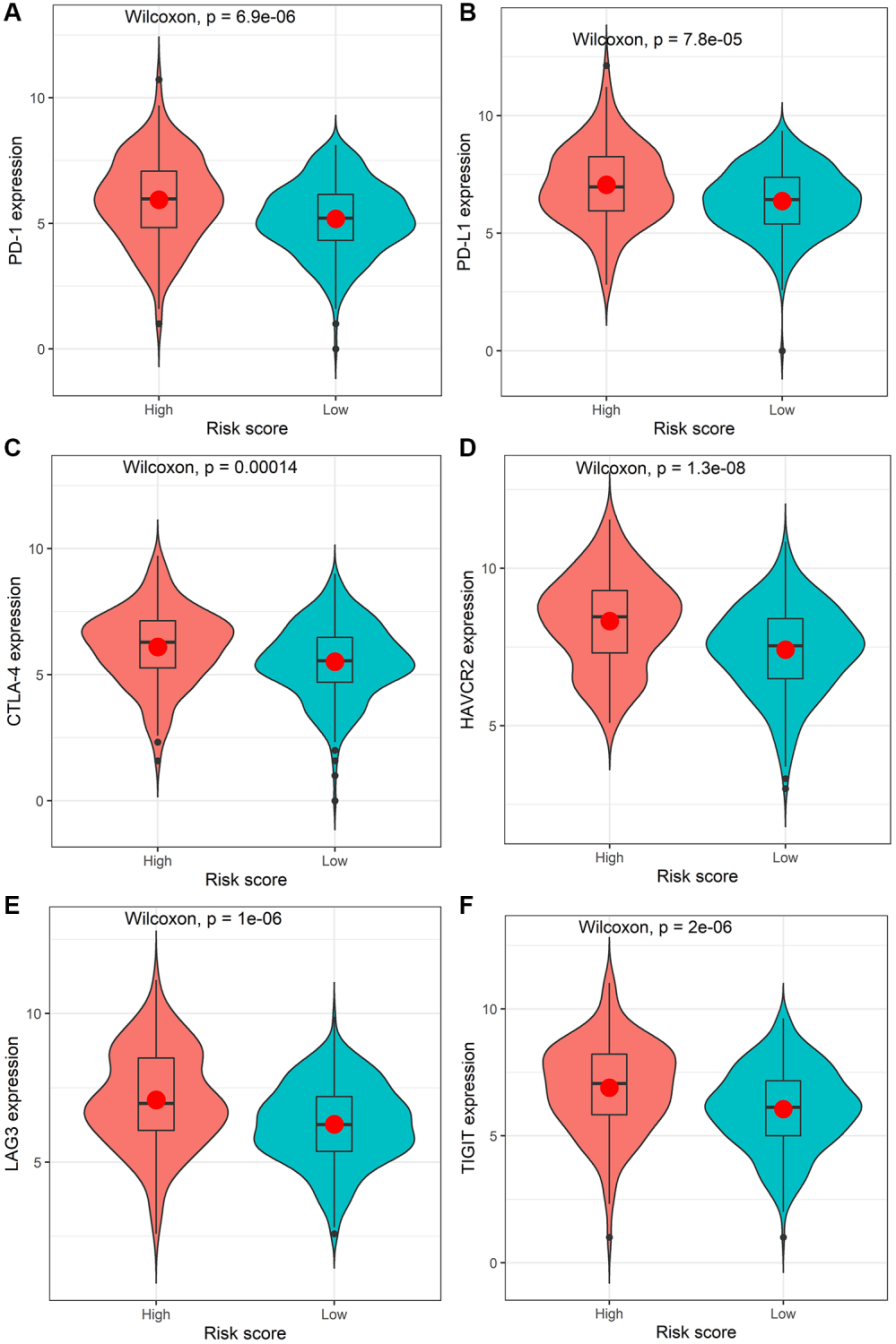
**Comparison of the expression levels of the immune checkpoints and their ligands between the high-risk score group and low-risk score group.** The expression of *PD-1* (**A**)*, PD-L1* (**B**)*, CTLA-4* (**C**)*, HAVCR2* (**D**)*, LAG3* (**E**), or *TIGIT* (**F**).

The mutation profiles of the high-risk group and low-risk group were plotted and compared using maftools R package. The top 20 significantly mutated genes were *APC, TP53, TTN, KRAS, SYNE1, MUC16, PIK3CA, FAT4, RYR2, OBSCN, ZFHX4, DNAH5, PCLO, CSMD3, ABCA13, DNAH11, LRP1B, FAT3, USH2A* and *CSMD1* (Figure 4D). Among them, the mutation rate of APC was significantly higher in the low-risk group (*P* = 0.000716, Figure 4E), while the mutation rate of *MUC16* was significantly higher in the high-risk group (*P* = 0.0154, Figure 4E). However, there was no significant association between the mutation status of *APC* or *MUC16* and patients’ OS (Figure 4F). Besides, the TMB value of each sample was calculated and visualized (median value of high-risk group: 2.66/MB; median value of low-risk group: 2/MB, Figure 4G). As shown in Figure 4H, the TMB value of patients in high-risk group was significantly higher than those in low-risk group (*P* = 0.00031). Moreover, the Wilcoxon test was applied to statistically compare the expression of immune checkpoint inhibitor targets between the two groups. The results revealed that the high-risk group had a significantly higher expression level (*P* < 0.001, Figure 5).

### The identified IRIGs as potential diagnosis biomarkers

**Figure 6:**
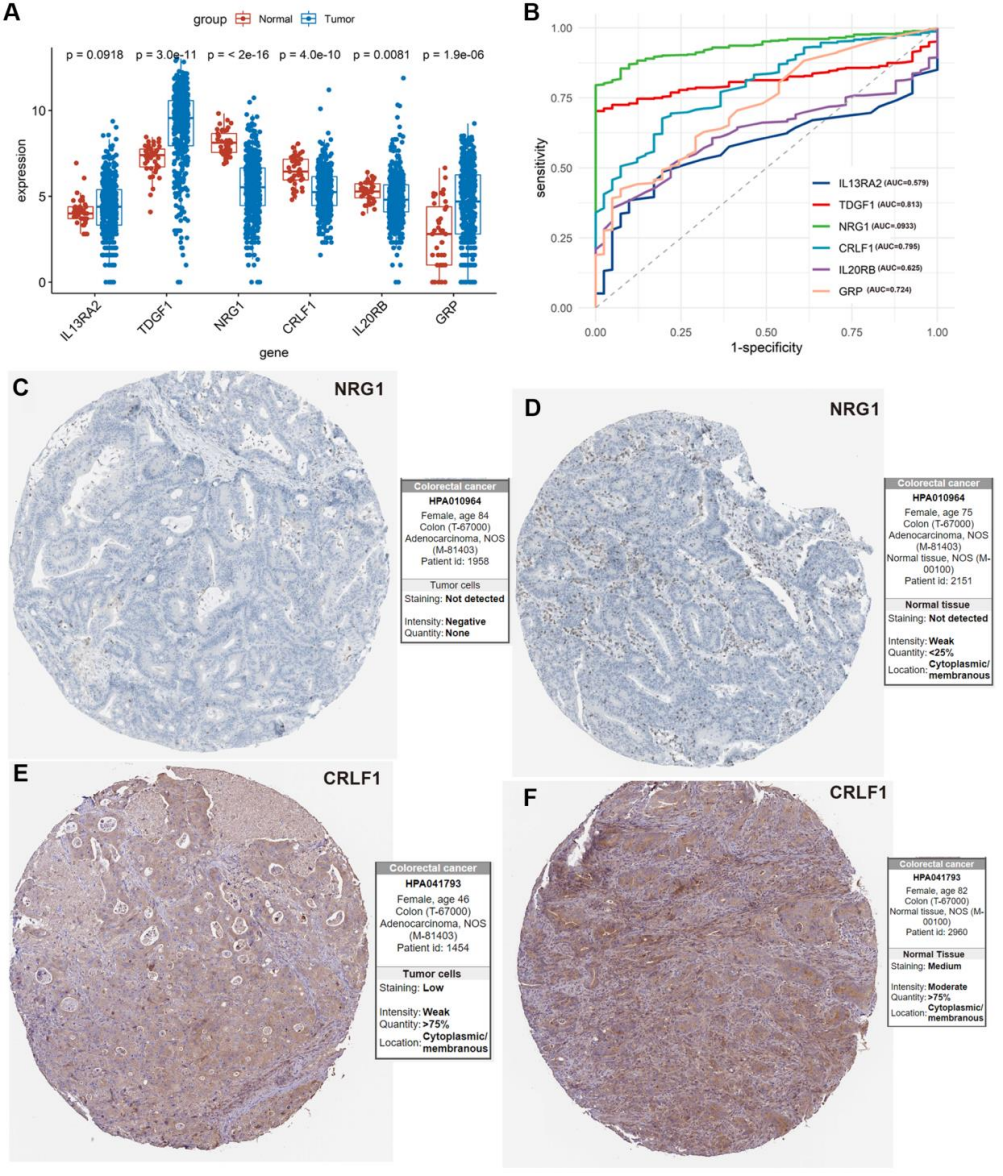
**Exploration of the 6 IRIGs as predictive diagnostic indicators based on the TCGA-COAD cohort.** (**A**) Comparison of the expressions of the IRIGs between tumor and normal samples using the Wilcoxon test. (**B**) Establishment of the predictive diagnosis model using the 6 IRIGs. (**C**–**F**) The comparison of protein expression of *NRG1* and *CRLF1* between tumor and normal tissues.

5 of 6 IRIGs were significantly differentially expressed between tumor and normal samples (*P* < 0.05, Figure 6A). Among them, *TDGF1*, and *GRP* were up-regulated in tumor samples, while *NRG1*, *CRLF1*, and *IL20RB* were down-regulated in tumor samples. The IRIGs were used to establish the prediction models with *RF* algorithm, and the AUC of each IRIG was calculated and plotted using the ggroc R package (*IL13RA2*: 0.579; *TDGF1*: 0.813; *NRG1*:0.933; *CRLF1*: 0.795; *IL20RB*: 0.625; *GRP*: 0.724, Figure 6B). Among them, *TDGF1* and *NRG1* revealed excellent diagnostic predictive performance (AUC >0.8). The distribution and expression of *CRLF1*, and *NRG1* at the protein level are shown in Figure 6C–6F, whereas *IL13RA2, TDGF1, IL20RB,* and *GRP* remained inaccessible in HPA.

## DISCUSSION

Extensive studies have been conducted to identify novel diagnosis, prognosis, and therapeutic targets for colon cancer [14–17]. However, more effective signatures needed to be explored. In this study, we tried to identify immune-related signatures for predicting the immunotherapeutic responsiveness, prognosis, and diagnosis of colon cancer. Firstly, immunotherapeutic responsiveness-related DEGs were identified by comparing the responders and non-responders in the anti-PD-L1 cohort, intersecting with IRGs, then obtained IRIGs. Then, 6 of these IRIGs (*TDGF1*, *IL13RA2*, *NRG1*, *CRLF1*, *GRP*, and *IL20RB*) were selected to establish an IRIRScore prognostic model. Among them, *TDGF, NRG1*, and *GRP* have been demonstrated as prognostic indicators for patients with colon cancer [18–20]. *TDGF1,* a member of the epidermal growth factor-cripto *FRL1* cryptic protein family, is involved in activating several different signaling pathways during embryonic development and cellular transformation, and could be a predictive marker for metachronous metastasis in patients with colorectal cancer [21]. Previous studies demonstrated that *IL13RA2* was overexpressed in various cancers, such as malignant gliomas [22], and thyroid Carcinoma [23]. In our study, the trend of the expression level of *IL13RA2* was also up-regulated in tumor samples compared with normal samples, though it was not significant (*P* = 0.0918, Figure 6A). *NRG1* can activate *HER3* to promote resistance in tumor cells [24]. *CRLF1*, as the target for miR-3065-3p, could promote the stemness and metastasis of colorectal cancer [25]. Besides, *CRLF1* regulates immune, inflammation, hematopoiesis, cell growth, and differentiation [26], which may relate to tumorigenesis. Moreover, the expression level of *CRLF1* was down-regulated in tumor samples compared with normal samples (*P* = 4.1e^−10^, Figure 6A, 6E and 6F). Gastrin Releasing Peptide (*GRP*), acting synergistically to promote cell proliferation, play an essential role in cancer development and are frequently over-expressed in various tumors [27]. According to the results of our study, *GRP* was also up-regulated in tumor samples compared with normal samples (*P* = 1.9e^−6^, Figure 6A). Previous studies have demonstrated that *IL20RB* was associated with the prognosis of various cancers, such as lung adenocarcinoma [28], pancreatic ductal adenocarcinoma [29], and pancreatic cancer [30].

The identified immune-related signatures in predicting prognosis and TNM staging were validated in multiple cohorts, and displayed robust capability. Moreover, a nomogram was established based on the IRIRScore and other clinical characters, and the C-index of the nomogram was remarkably higher than the IRIRScore model. These findings may be helpful for the prognosis of patients with colon cancer. Additionally, we found that patients in the high-risk group had significantly higher expression level of immune checkpoint inhibitor targets than those in the low-risk group (Figure 5), suggesting that high-risk patients may respond better to immune checkpoint inhibitors targeting *PD-1, PD-L1, CTLA-4, HAVCR2, LAG3,* or *TIGIT.* Besides, higher TMB value (Figure 4H), more instability of the microsatellite status (Figure 3E), or a higher mutation rate of *MUC16* (Figure 4E) are significantly associated with patients in the high-risk group, suggesting that those patients are more likely to benefit from immunotherapy. However, patients in the high-risk group exhibit a low mutation rate of *APC* (Figure 4E), suggesting that *APC* may be a tumor suppressor gene. In addition, patients with sensitive immunotherapeutic responsiveness may have a significantly better OS (Supplementary Figure 3C, 3E).

Furthermore, we investigated the 6 prognostic IRIGs in the diagnostic prediction of colon cancer. Among them, *TDGF1* and *NRG1* could be used as an independent diagnostic indicator and reveal excellent performance (Figure 6B, AUC >0.8).

In conclusion, our research provided new insights into immune-related immunotherapeutic responsiveness, prognosis, and diagnosis of colon cancer. However, there are still some limitations to our study. First, the datasets used in our study are based on public databases, and more experimental data were needed to verify our findings. Moreover, the molecular function and mechanism of the prognostic IRIGs need to be further investigated.

## Supplementary Materials

Supplementary Figures

Supplementary Tables 1-3 and 5

Supplementary Table 4

## References

[1] Siegel RL, Miller KD, Fuchs HE, Jemal A. Cancer statistics, 2022. CA Cancer J Clin. 2022; 72:7–33. 10.3322/caac.21708 35020204

[2] Lei X, Lei Y, Li JK, Du WX, Li RG, Yang J, Li J, Li F, Tan HB. Immune cells within the tumor microenvironment: Biological functions and roles in cancer immunotherapy. Cancer Lett. 2020; 470:126–33. 10.1016/j.canlet.2019.11.009 31730903

[3] Cao L, Li T, Ba Y, Chen E, Yang J, Zhang H. Exploring Immune-Related Prognostic Signatures in the Tumor Microenvironment of Colon Cancer. Front Genet. 2022; 13:801484. 10.3389/fgene.2022.801484 35281839 PMC8907673

[4] Liu J, Huang X, Liu H, Wei C, Ru H, Qin H, Lai H, Meng Y, Wu G, Xie W, Mo X, Johnson CH, Zhang Y, Tang W. Immune landscape and prognostic immune-related genes in KRAS-mutant colorectal cancer patients. J Transl Med. 2021; 19:27. 10.1186/s12967-020-02638-9 33413474 PMC7789428

[5] Li X, Wen D, Li X, Yao C, Chong W, Chen H. Identification of an Immune Signature Predicting Prognosis Risk and Lymphocyte Infiltration in Colon Cancer. Front Immunol. 2020; 11:1678. 10.3389/fimmu.2020.01678 33013820 PMC7497441

[6] Franke AJ, Skelton WP, Starr JS, Parekh H, Lee JJ, Overman MJ, Allegra C, George TJ. Immunotherapy for Colorectal Cancer: A Review of Current and Novel Therapeutic Approaches. J Natl Cancer Inst. 2019; 111:1131–41. 10.1093/jnci/djz093 31322663 PMC6855933

[7] Lichtenstern CR, Ngu RK, Shalapour S, Karin M. Immunotherapy, Inflammation and Colorectal Cancer. Cells. 2020; 9:618. 10.3390/cells9030618 32143413 PMC7140520

[8] Mariathasan S, Turley SJ, Nickles D, Castiglioni A, Yuen K, Wang Y, Kadel EE III, Koeppen H, Astarita JL, Cubas R, Jhunjhunwala S, Banchereau R, Yang Y, et al. TGFβ attenuates tumour response to PD-L1 blockade by contributing to exclusion of T cells. Nature. 2018; 554:544–8. 10.1038/nature25501 29443960 PMC6028240

[9] Yoshihara K, Shahmoradgoli M, Martínez E, Vegesna R, Kim H, Torres-Garcia W, Treviño V, Shen H, Laird PW, Levine DA, Carter SL, Getz G, Stemke-Hale K, et al. Inferring tumour purity and stromal and immune cell admixture from expression data. Nat Commun. 2013; 4:2612. 10.1038/ncomms3612 24113773 PMC3826632

[10] Robinson MD, McCarthy DJ, Smyth GK. edgeR: a Bioconductor package for differential expression analysis of digital gene expression data. Bioinformatics. 2010; 26:139–40. 10.1093/bioinformatics/btp616 19910308 PMC2796818

[11] Yu G, Wang LG, Han Y, He QY. clusterProfiler: an R package for comparing biological themes among gene clusters. OMICS. 2012; 16:284–7. 10.1089/omi.2011.0118 22455463 PMC3339379

[12] Newman AM, Liu CL, Green MR, Gentles AJ, Feng W, Xu Y, Hoang CD, Diehn M, Alizadeh AA. Robust enumeration of cell subsets from tissue expression profiles. Nat Methods. 2015; 12:453–7. 10.1038/nmeth.3337 25822800 PMC4739640

[13] Mayakonda A, Lin DC, Assenov Y, Plass C, Koeffler HP. Maftools: efficient and comprehensive analysis of somatic variants in cancer. Genome Res. 2018; 28:1747–56. 10.1101/gr.239244.118 30341162 PMC6211645

[14] Cao L, Chen E, Zhang H, Ba Y, Yan B, Li T, Yang J. Construction of a novel methylation-related prognostic model for colorectal cancer based on microsatellite status. J Cell Biochem. 2021; 122:1781–90. 10.1002/jcb.30131 34397105

[15] Cai C, Peng Y, Shen E, Wan R, Gao L, Gao Y, Zhou Y, Huang Q, Chen Y, Liu P, Guo C, Feng Z, Zhang X, et al. Identification of tumour immune infiltration-associated snoRNAs (TIIsno) for predicting prognosis and immune landscape in patients with colon cancer via a TIIsno score model. EBioMedicine. 2022; 76:103866. 10.1016/j.ebiom.2022.103866 35144219 PMC8844792

[16] Cui Z, Sun G, Bhandari R, Lu J, Zhang M, Bhandari R, Sun F, Liu Z, Zhao S. Comprehensive Analysis of Glycolysis-Related Genes for Prognosis, Immune Features, and Candidate Drug Development in Colon Cancer. Front Cell Dev Biol. 2021; 9:684322. 10.3389/fcell.2021.684322 34422808 PMC8377503

[17] Zhou R, Zhang J, Zeng D, Sun H, Rong X, Shi M, Bin J, Liao Y, Liao W. Immune cell infiltration as a biomarker for the diagnosis and prognosis of stage I-III colon cancer. Cancer Immunol Immunother. 2019; 68:433–42. 10.1007/s00262-018-2289-7 30564892 PMC6426802

[18] Sun YL, Zhang Y, Guo YC, Yang ZH, Xu YC. A Prognostic Model Based on the Immune-related Genes in Colon Adenocarcinoma. Int J Med Sci. 2020; 17:1879–96. 10.7150/ijms.45813 32788867 PMC7415395

[19] Xu J, Dai S, Yuan Y, Xiao Q, Ding K. A Prognostic Model for Colon Cancer Patients Based on Eight Signature Autophagy Genes. Front Cell Dev Biol. 2020; 8:602174. 10.3389/fcell.2020.602174 33324651 PMC7726244

[20] Li C, Shen Z, Zhou Y, Yu W. Independent prognostic genes and mechanism investigation for colon cancer. Biol Res. 2018; 51:10. 10.1186/s40659-018-0158-7 29653552 PMC5897983

[21] Miyoshi N, Ishii H, Mimori K, Sekimoto M, Doki Y, Mori M. TDGF1 is a novel predictive marker for metachronous metastasis of colorectal cancer. Int J Oncol. 2010; 36:563–8. 10.3892/ijo_00000530 20126975

[22] Zeng J, Zhang J, Yang YZ, Wang F, Jiang H, Chen HD, Wu HY, Sai K, Hu WM. IL13RA2 is overexpressed in malignant gliomas and related to clinical outcome of patients. Am J Transl Res. 2020; 12:4702–14. 32913543 PMC7476143

[23] Chong ST, Tan KM, Kok CYL, Guan SP, Lai SH, Lim C, Hu J, Sturgis C, Eng C, Lam PYP, Ngeow J. IL13RA2 Is Differentially Regulated in Papillary Thyroid Carcinoma vs Follicular Thyroid Carcinoma. J Clin Endocrinol Metab. 2019; 104:5573–84. 10.1210/jc.2019-00040 31290966

[24] Zhang Z, Karthaus WR, Lee YS, Gao VR, Wu C, Russo JW, Liu M, Mota JM, Abida W, Linton E, Lee E, Barnes SD, Chen HA, et al. Tumor Microenvironment-Derived NRG1 Promotes Antiandrogen Resistance in Prostate Cancer. Cancer Cell. 2020; 38:279–96.e9. 10.1016/j.ccell.2020.06.005 32679108 PMC7472556

[25] Li Y, Xun J, Wang B, Ma Y, Zhang L, Yang L, Gao R, Guan J, Liu T, Gao H, Wang X, Zhang Q. miR-3065-3p promotes stemness and metastasis by targeting CRLF1 in colorectal cancer. J Transl Med. 2021; 19:429. 10.1186/s12967-021-03102-y 34656128 PMC8520297

[26] Crisponi L, Buers I, Rutsch F. CRLF1 and CLCF1 in Development, Health and Disease. Int J Mol Sci. 2022; 23:992. 10.3390/ijms23020992 35055176 PMC8780587

[27] Palmioli A, Nicolini G, Tripodi F, Orsato A, Ceresa C, Donzelli E, Arici M, Coccetti P, Rocchetti M, La Ferla B, Airoldi C. Targeting GRP receptor: Design, synthesis and preliminary biological characterization of new non-peptide antagonists of bombesin. Bioorg Chem. 2021; 109:104739. 10.1016/j.bioorg.2021.104739 33626451

[28] Zhang M, Zhu K, Pu H, Wang Z, Zhao H, Zhang J, Wang Y. An Immune-Related Signature Predicts Survival in Patients With Lung Adenocarcinoma. Front Oncol. 2019; 9:1314. 10.3389/fonc.2019.01314 31921619 PMC6914845

[29] Wang W, Yan L, Guan X, Dong B, Zhao M, Wu J, Tian X, Hao C. Identification of an Immune-Related Signature for Predicting Prognosis in Patients With Pancreatic Ductal Adenocarcinoma. Front Oncol. 2021; 10:618215. 10.3389/fonc.2020.618215 33718118 PMC7945593

[30] Liu B, Fu T, He P, Du C, Xu K. Construction of a five-gene prognostic model based on immune-related genes for the prediction of survival in pancreatic cancer. Biosci Rep. 2021; 41:BSR20204301. 10.1042/BSR20204301 34143198 PMC8252190

