# Supplementary material for: Exploring immune-related signatures for predicting immunotherapeutic responsiveness, prognosis, and diagnosis of patients with colon cancer
Source: Aging (Albany NY). 2022 Jun 20;14(12):5131–52. doi: 10.18632/aging.204134 (PMC9271306; doi:10.18632/aging.204134)
Supplement: Supplementary Figures [file aging-14-204134-s001.pdf]

SUPPLEMENTARY FIGURES

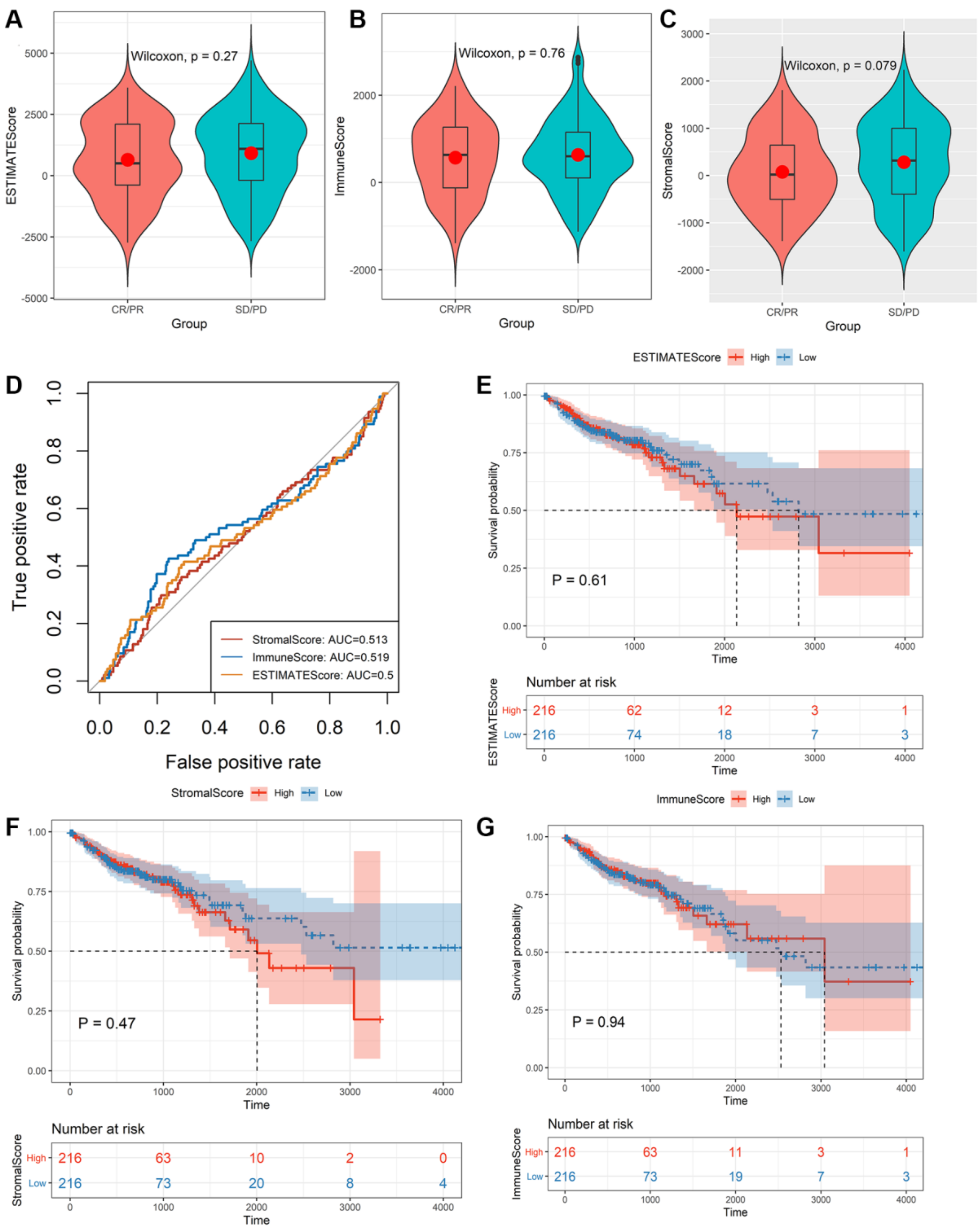

Supplementary Figure 1. Construction and assessment of estimate-stromal-immune scores model. (**A**–**C**) Comparison of the differences of the estimate-stromal-immune scores between responders and non-responders using Wilcoxon test. (**D**) The AUCs of the estimate-stromal-immune scores model. (**E**–**G**) Kaplan-Meier curves analyses of the association between the scores (estimate, stromal, or immune) and patients' OS.

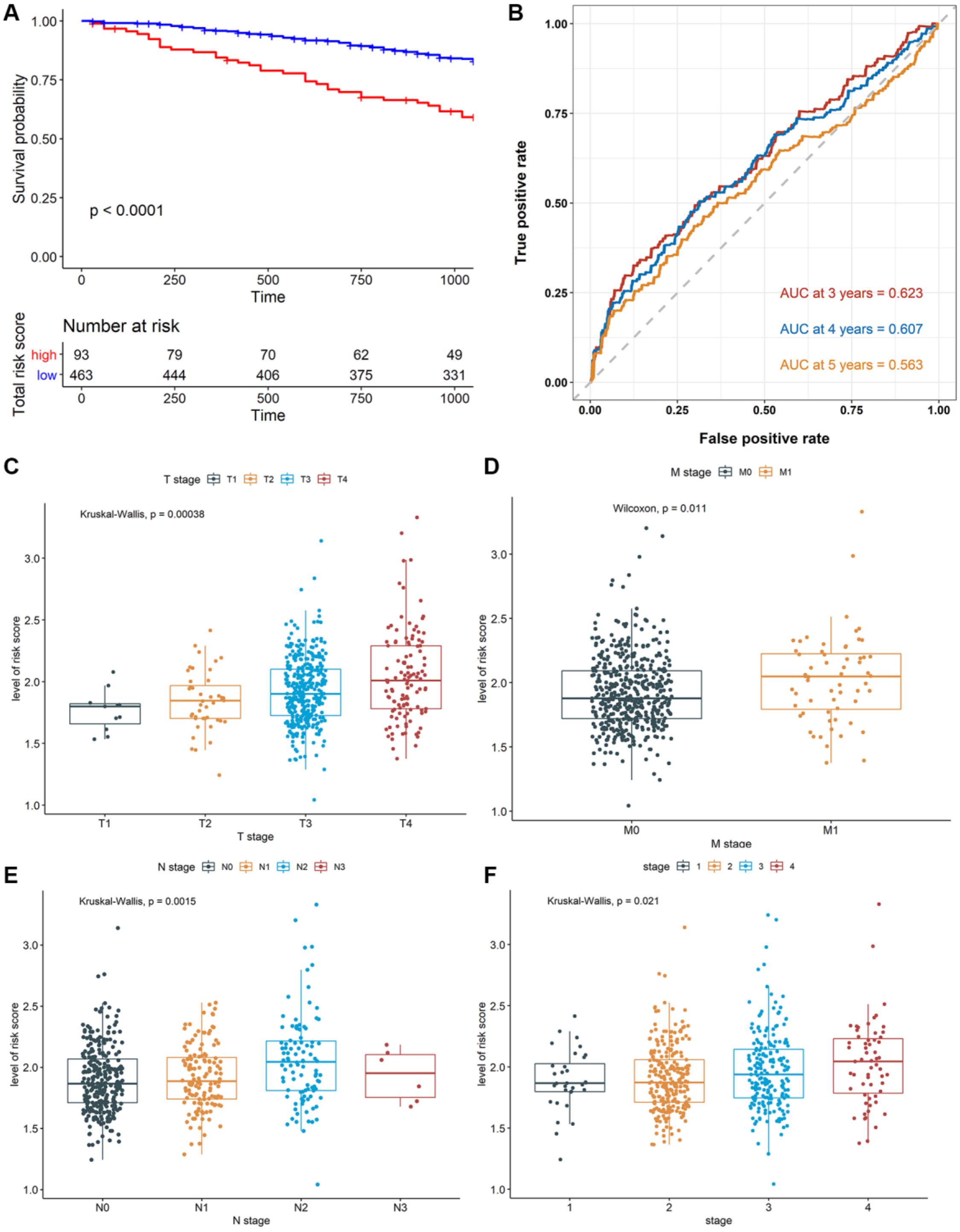

Supplementary Figure 2. Validation of the identified immune-related prognostic signatures using GSE39582. (**A**) Kaplan-Meier curve analysis. (**B**) Time-dependent ROC curve analysis. (**C**–**F**) Comparison of the association between the IRIRScore of the colon cancer patients and the clinical-pathological characteristics, including (**C**) T stages, (**D**) M stages, (**E**) N stages and (**F**) advanced pathological stages.

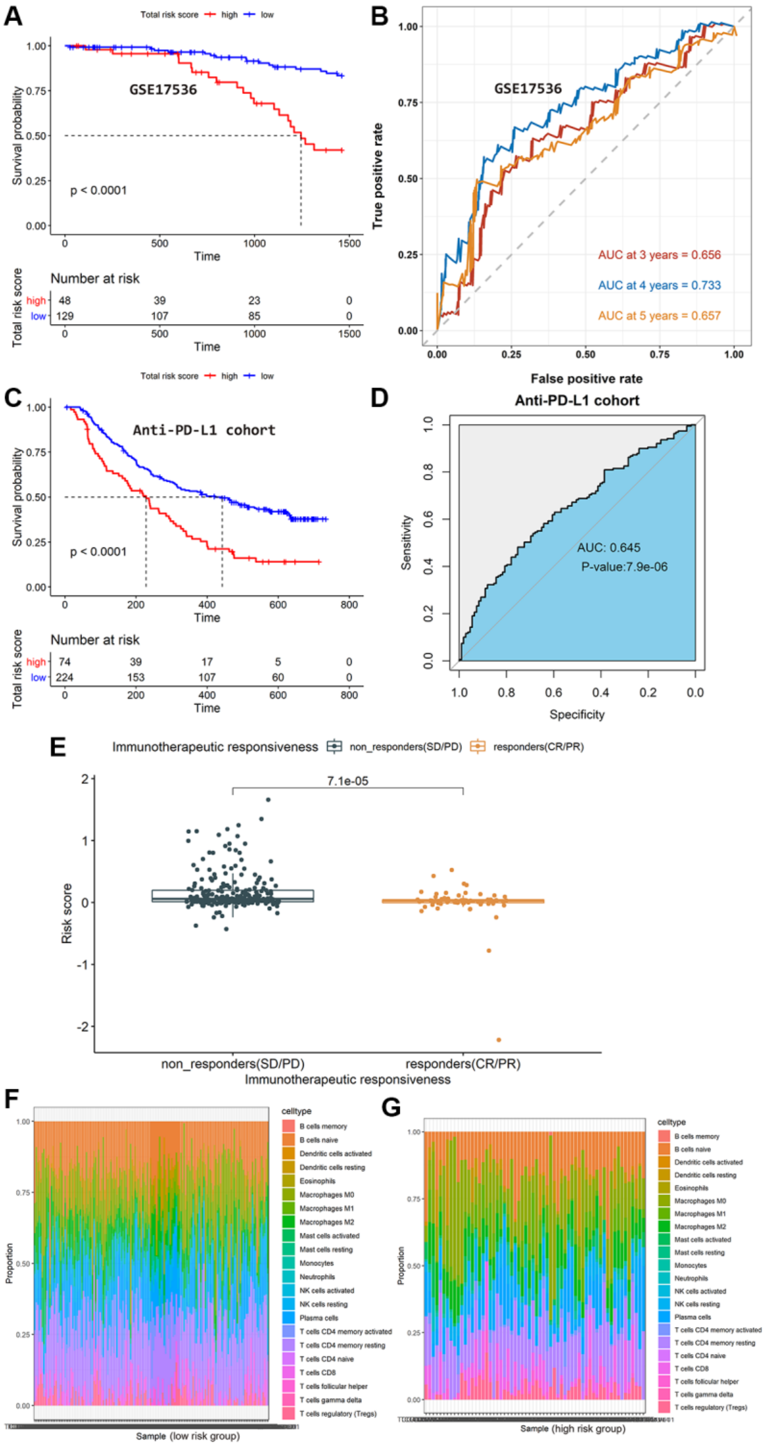

Supplementary Figure 3. Validation of the identified immune-related prognostic signatures using GSE17536 and anti-PD-L1 cohort. (**A**) Kaplan-Meier curve analysis using GSE17536. (**B**) Time-dependent ROC curve analysis using 17536. (**C**) Kaplan-Meier curve analysis using anti-PD-L1 cohort. (**D**) Time-dependent ROC curve analysis using anti-PD-L1 cohort. (**E**) Comprising the difference in the risk score between responders and non-responders in anti-PD-L1 cohort. (**F**–**G**) The profiles of relative proportions of immune infiltration. (**F**) Patients in low-risk group, (**G**) patients in high-risk group.

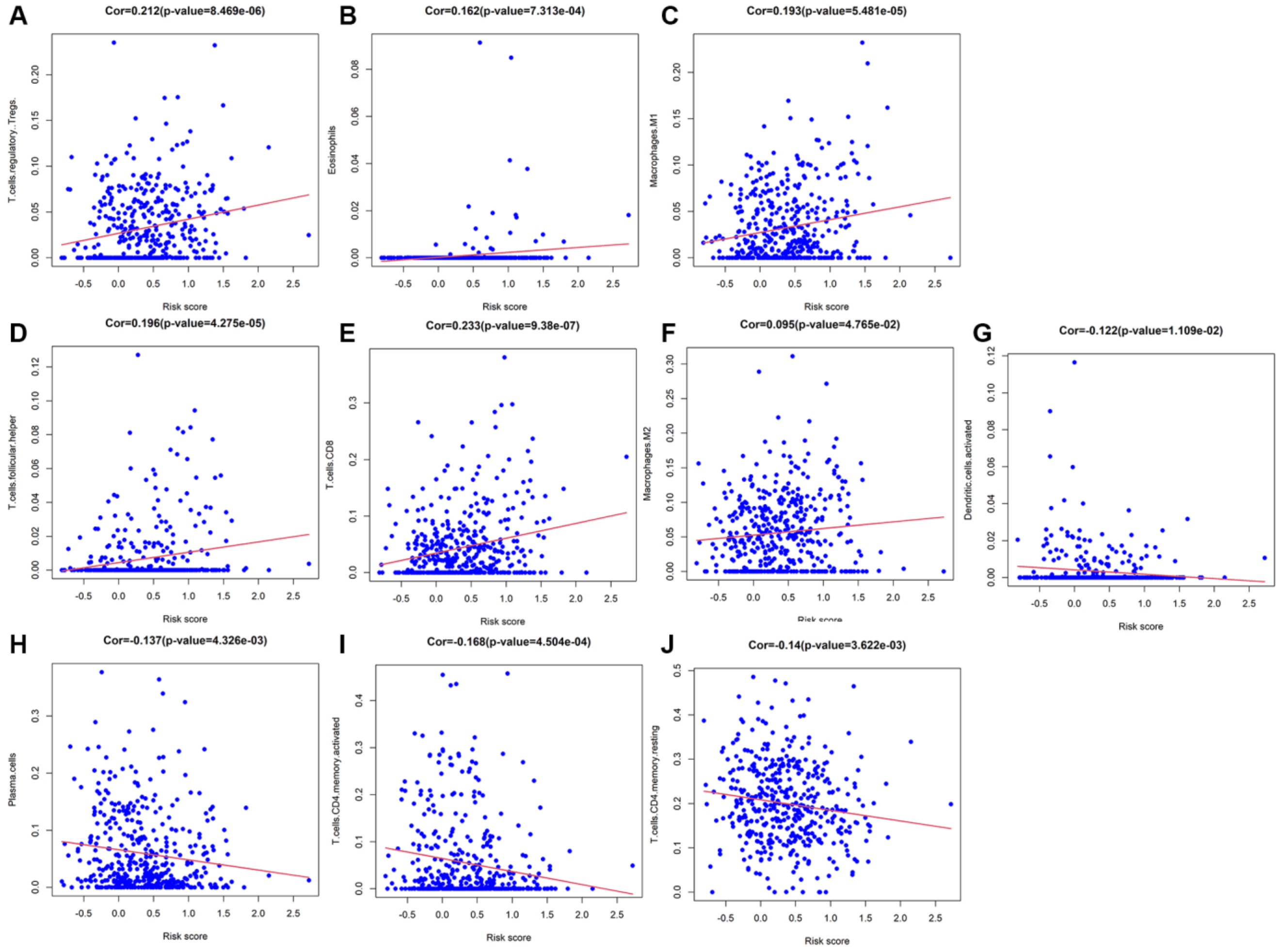

Supplementary Figure 4. The correlation between IRIRScore and the proportions of immune cells infiltrating, including (**A**) T cells regulatory (Tregs), (**B**) Eosinophils, (**C**) Macrophages M1, (**D**) T cells follicular helper, (**E**) T cells CD8, (**F**) Macrophages M2, (**G**) Dendritic cells activated, (**H**) Plasma cells, (**I**) T cells CD4 memory activated, (**J**) T cells CD4 memory resting.
