# Supplementary material for: Exploring immune-related signatures for predicting immunotherapeutic responsiveness, prognosis, and diagnosis of patients with colon cancer
Source: Aging (Albany NY). 2022 Jun 20;14(12):5131–52. doi: 10.18632/aging.204134 (PMC9271306; doi:10.18632/aging.204134)
Supplement: Supplementary Tables 1-3 and 5 [file aging-14-204134-s002.pdf]

SUPPLEMENTARY TABLES

Supplementary Table 1. The detailed information of TCGA-COAD cohort.

	Tumor	Normal	Overall
	**(*N* = 432)**	**(*N* = 39)**	**(*N* = 471)**
**Gender**			
Female	200 (46.3%)	20 (51.3%)	220 (46.7%)
Male	232 (53.7%)	19 (48.7%)	251 (53.3%)
**Age**			
≤60	136 (31.5%)	9 (23.1%)	145 (30.8%)
>60	296 (68.5%)	30 (76.9%)	326 (69.2%)
**AJCC-Stage**			
I	73 (16.9%)	4 (10.3%)	77 (16.3%)
II	166 (38.4%)	21 (53.8%)	187 (39.7%)
III	122 (28.2%)	6 (15.4%)	128 (27.2%)
IV	60 (13.9%)	7 (17.9%)	67 (14.2%)
Missing	11 (2.5%)	1 (2.6%)	12 (2.5%)
**AJCC-T**			
T1	11 (2.5%)	0 (0%)	11 (2.3%)
T2	75 (17.4%)	5 (12.8%)	80 (17.0%)
T3	296 (68.5%)	28 (71.8%)	324 (68.8%)
T4	49 (11.3%)	6 (15.4%)	55 (11.7%)
Missing	1 (0.2%)	0 (0%)	1 (0.2%)
**AJCC-M**			
M0	318 (73.6%)	25 (64.1%)	343 (72.8%)
M1	60 (13.9%)	7 (17.9%)	67 (14.2%)
MX	47 (10.9%)	6 (15.4%)	53 (11.3%)
Missing	7 (1.6%)	1 (2.6%)	8 (1.7%)
**AJCC-N**			
N0	254 (58.8%)	27 (69.2%)	281 (59.7%)
N1	100 (23.1%)	7 (17.9%)	107 (22.7%)
N2	78 (18.1%)	5 (12.8%)	83 (17.6%)

Supplementary Table 2. The detailed information of GSE39582.

	Alive	Death	Overall
	**(*N* = 369)**	**(*N* = 187)**	**(*N* = 556)**
**Gender**			
Female	175 (47.4%)	74 (39.6%)	249 (44.8%)
Male	194 (52.6%)	113 (60.4%)	307 (55.2%)
**Age**			
≤60	115 (31.2%)	42 (22.5%)	157 (28.2%)
>60	254 (68.8%)	145 (77.5%)	399 (71.8%)
**AJCC-Stage**			
0	3 (0.8%)	1 (0.5%)	4 (0.7%)
1	27 (7.3%)	5 (2.7%)	32 (5.8%)
2	183 (49.6%)	75 (40.1%)	258 (46.4%)
3	136 (36.9%)	67 (35.8%)	203 (36.5%)

4	20 (5.4%)	39 (20.9%)	59 (10.6%)
**AJCC-T**			
N/A	8 (2.2%)	12 (6.4%)	20 (3.6%)
T0	1 (0.3%)	0 (0%)	1 (0.2%)
T1	10 (2.7%)	1 (0.5%)	11 (2.0%)
T2	36 (9.8%)	8 (4.3%)	44 (7.9%)
T3	249 (67.5%)	111 (59.4%)	360 (64.7%)
T4	63 (17.1%)	54 (28.9%)	117 (21.0%)
Tis	2 (0.5%)	1 (0.5%)	3 (0.5%)
**AJCC-M**			
M0	340 (92.1%)	134 (71.7%)	474 (85.3%)
M1	20 (5.4%)	40 (21.4%)	60 (10.8%)
MX	1 (0.3%)	1 (0.5%)	2 (0.4%)
N/A	8 (2.2%)	12 (6.4%)	20 (3.6%)
**AJCC-N**			
N/A	8 (2.2%)	12 (6.4%)	20 (3.6%)
N+	1 (0.3%)	5 (2.7%)	6 (1.1%)
N0	214 (58.0%)	81 (43.3%)	295 (53.1%)
N1	91 (24.7%)	40 (21.4%)	131 (23.6%)
N2	52 (14.1%)	46 (24.6%)	98 (17.6%)
N3	3 (0.8%)	3 (1.6%)	6 (1.1%)

Abbreviation: AJCC: American Joint Committee on Cancer.

Supplementary Table 3. The detailed information of GSE17536.

	**Alive**	**Death**	**Overall**
	**(*N* = 73)**	**(*N* = 104)**	**(*N* = 177)**
**Gender**			
Female	32 (43.8%)	49 (47.1%)	81 (45.8%)
Male	41 (56.2%)	55 (52.9%)	96 (54.2%)
**Age**			
≤60	26 (35.6%)	33 (31.7%)	59 (33.3%)
>60	47 (64.4%)	71 (68.3%)	118 (66.7%)
**AJCC-stage**			
1	4 (5.5%)	20 (19.2%)	24 (13.6%)
2	12 (16.4%)	45 (43.3%)	57 (32.2%)
3	25 (34.2%)	32 (30.8%)	57 (32.2%)
4	32 (43.8%)	7 (6.7%)	39 (22.0%)

Abbreviation: AJCC: American Joint Committee on Cancer.

Supplementary Table 5. The detailed information of IRIGs.

Gene Name	logFC	logCPM	LR	*P* value	FDR	change
NPY	8.07268	1.07696	122.324	1.96E-28	5.54E-25	UP
SEMG2	4.07083	3.58072	57.4661	3.44E-14	1.39E-11	UP
ALB	−5.7925	8.50241	54.1559	1.85E-13	6.58E-11	DOWN
SCGB3A1	3.0018	0.24711	48.5146	3.28E-12	9.02E-10	UP
IL13RA2	1.69046	1.61056	43.4311	4.39E-11	1.03E-08	UP
HAMP	−2.7598	2.91445	40.748	1.73E-10	3.61E-08	DOWN
ORM1	−5.4108	7.03914	40.1051	2.41E-10	4.89E-08	DOWN
PRL	2.7778	−3.3736	39.2792	3.67E-10	6.96E-08	UP
SEMG1	3.56408	4.18769	38.7377	4.85E-10	8.81E-08	UP
HRG	−5.9652	3.80736	37.9741	7.17E-10	1.29E-07	DOWN
KLRC2	1.30054	1.04129	34.6247	4.00E-09	5.86E-07	UP
SAA2	−3.3183	3.34611	34.1954	4.98E-09	7.11E-07	DOWN
DMBT1	2.09005	5.80462	34.0219	5.45E-09	7.74E-07	UP
IL17RB	1.08385	2.40642	33.9975	5.52E-09	7.80E-07	UP
FGF9	1.40722	−0.0577	33.3619	7.65E-09	1.04E-06	UP
GLP1R	1.66604	−1.0846	33.2311	8.18E-09	1.11E-06	UP
IL1RL1	−1.262	3.07036	32.1206	1.45E-08	1.85E-06	DOWN
SAA1	−2.7309	4.41777	30.667	3.06E-08	3.58E-06	DOWN
SSTR2	1.05883	1.12259	29.3184	6.14E-08	6.65E-06	UP
KLRC3	1.13029	0.34016	28.7809	8.10E-08	8.60E-06	UP
KNG1	−5.0457	3.16261	27.9993	1.21E-07	1.25E-05	DOWN
TDGF1	−3.0484	−2.3637	27.841	1.32E-07	1.34E-05	DOWN
FGA	−5.7725	7.00837	27.3696	1.68E-07	1.63E-05	DOWN
VTN	−2.6408	2.36105	27.0228	2.01E-07	1.89E-05	DOWN
LEP	2.07134	0.97879	26.6754	2.41E-07	2.21E-05	UP
FCN2	−3.8681	−1.2927	25.4887	4.45E-07	3.82E-05	DOWN
INHBC	−2.8824	−1.992	25.196	5.18E-07	4.40E-05	DOWN
RXRG	−2.3664	−1.4136	24.6196	6.98E-07	5.65E-05	DOWN
NRG1	−1.4294	3.85232	23.9393	9.94E-07	7.79E-05	DOWN
PRLR	1.25391	3.20478	23.5388	1.22E-06	9.43E-05	UP
RXFP2	−4.867	−0.0549	22.0335	2.68E-06	0.00018	DOWN
GDF5	−1.7395	−1.5743	21.2493	4.03E-06	0.00025	DOWN
SERPINA3	−1.871	7.13183	21.1975	4.14E-06	0.00026	DOWN
MASP2	−4.8815	−1.1652	20.8945	4.85E-06	0.0003	DOWN
ANGPTL3	−5.3865	1.8288	20.8437	4.98E-06	0.0003	DOWN
CRLF1	−1.2931	1.51951	20.1631	7.11E-06	0.0004	DOWN
CCL16	−1.8697	−2.445	19.9142	8.10E-06	0.00045	DOWN
ADCYAP1R1	1.06672	−0.6269	19.2477	1.15E-05	0.0006	UP
DEFA5	5.28237	−2.572	18.9598	1.34E-05	0.00067	UP
APOH	−6.1389	2.79076	18.8944	1.38E-05	0.00069	DOWN
CXCL9	1.12379	4.63729	18.5748	1.63E-05	0.00079	UP
GCGR	−1.5663	−0.9993	18.5641	1.64E-05	0.00079	DOWN
HTR3E	1.67214	−2.806	18.2836	1.90E-05	0.00088	UP
IL36G	−1.9226	0.04119	18.2516	1.94E-05	0.0009	DOWN
IL5RA	−1.3291	−0.0271	16.9115	3.92E-05	0.00157	DOWN
MTNR1B	1.96453	−0.1287	16.5586	4.72E-05	0.00183	UP
PLA2G2A	−1.4746	6.56759	16.193	5.72E-05	0.00214	DOWN
PPBP	1.22015	1.75033	16.1397	5.88E-05	0.00219	UP
STAB2	−1.4416	2.45814	14.9072	0.00011	0.00362	DOWN
IL11	−1.0718	−0.5759	14.8993	0.00011	0.00363	DOWN
OPRD1	2.0922	−3.7315	14.7374	0.00012	0.00388	UP
NR1I2	−1.3061	−0.7679	14.6061	0.00013	0.00408	DOWN
PCSK2	1.36054	−1.7084	14.5353	0.00014	0.00416	UP
CCL14	−1.0164	0.53176	14.4263	0.00015	0.00436	DOWN

HNF4A	−1.6919	1.32014	13.7062	0.00021	0.00583	DOWN
CGA	−3.3334	1.01221	13.4856	0.00024	0.00638	DOWN
PROC	−1.0469	0.93374	13.4168	0.00025	0.00656	DOWN
NOS2	1.01727	1.82741	13.1488	0.00029	0.00727	UP
ANGPT4	−1.5943	−1.3712	13.0615	0.0003	0.00751	DOWN
TRHR	2.33717	−3.8388	13.0064	0.00031	0.00766	UP
NOS1	−1.4171	−0.5483	12.8839	0.00033	0.008	DOWN
IFNG	1.0106	−1.8537	12.8652	0.00033	0.00807	UP
TMPRSS6	−1.3025	0.39655	12.7854	0.00035	0.00833	DOWN
GFAP	1.55922	−2.0394	12.4555	0.00042	0.00958	UP
CRHR1	1.67197	−3.9325	12.4378	0.00042	0.00963	UP
MC4R	1.16876	−1.6198	12.2442	0.00047	0.01036	UP
AGTR2	−2.3464	−1.3464	12.2296	0.00047	0.01041	DOWN
IL20RB	−1.1126	3.46575	12.0512	0.00052	0.01111	DOWN
PRTN3	−2.4749	−3.6688	11.645	0.00064	0.01288	DOWN
SCTR	−2.445	−0.92	11.6004	0.00066	0.01309	DOWN
CRHR2	−1.6053	−2.3542	11.2189	0.00081	0.01529	DOWN
LECT2	−5.8868	−2.1614	10.7966	0.00102	0.01801	DOWN
KIR3DL1	1.25468	−2.4293	10.6348	0.00111	0.01918	UP
CHGA	−2.3938	1.94464	10.2617	0.00136	0.02219	DOWN
IL36A	−2.0358	−3.1271	10.2502	0.00137	0.02228	DOWN
EPGN	1.09948	1.28689	10.1555	0.00144	0.02301	UP
KIR2DL3	1.14603	−2.5951	10.0633	0.00151	0.02386	UP
CCL21	−1.1071	3.14221	9.19801	0.00242	0.0337	DOWN
NPPA	2.94851	−4.0574	9.18006	0.00245	0.03389	UP
AZU1	−1.1304	−3.6622	9.02722	0.00266	0.03611	DOWN
GRP	−1.1081	−1.1179	8.84953	0.00293	0.0388	DOWN
GDF2	−3.1711	−3.4948	8.11006	0.0044	0.05169	DOWN
IL20	−1.5832	−1.4149	7.75751	0.00535	0.05892	DOWN
REG1A	−2.1373	−1.4859	7.59447	0.00585	0.06245	DOWN
TRH	1.0349	−4.1896	7.14708	0.00751	0.07464	UP
CRP	−2.4574	4.84234	7.14605	0.00751	0.07466	DOWN
LEFTY1	−1.1188	−3.2202	7.10331	0.00769	0.07584	DOWN
IFNA7	−4.6106	−2.5514	6.89712	0.00863	0.08167	DOWN
SLURP1	1.05007	−2.4612	6.73495	0.00945	0.0869	UP
DEFA3	−1.6148	−2.5118	6.56553	0.0104	0.09269	DOWN
IFNA10	−4.477	−2.7021	6.3096	0.01201	0.10241	DOWN
SST	−2.1199	3.65444	6.06229	0.01381	0.11247	DOWN
IFNA13	−2.2656	−4.1362	5.73674	0.01661	0.12666	DOWN
GCG	−6.9615	−0.3735	5.71844	0.01679	0.12747	DOWN
GHRHR	−1.6539	−1.4075	5.62295	0.01773	0.13218	DOWN
IFNA4	−4.3846	−3.2965	5.43798	0.0197	0.14048	DOWN
FGF23	−2.0547	−3.3676	5.33392	0.02091	0.14594	DOWN
CSH2	−2.3671	−3.0927	5.24551	0.022	0.15099	DOWN
NR1I3	−1.1059	−1.8725	5.20664	0.0225	0.15324	DOWN
IFNA21	−4.5568	−2.1071	5.17558	0.02291	0.15507	DOWN
CRH	−1.4468	1.14366	5.0405	0.02476	0.1628	DOWN
HTR3C	−1.3177	−2.9042	4.99308	0.02545	0.16588	DOWN
BMP10	−1.0623	−3.0794	4.75552	0.0292	0.18081	DOWN
KIR2DL1	1.02773	−3.2495	4.4789	0.03432	0.20078	UP
IL17A	1.14292	−2.8353	4.37489	0.03647	0.2088	UP
AMELX	1.3256	−3.9947	4.35373	0.03693	0.21063	UP
NTS	−1.1096	4.24173	4.13124	0.0421	0.22775	DOWN
FSHR	−1.0155	−3.5468	3.86795	0.04922	0.2511	DOWN
